# The requirement for the LysR-type regulator PtrA for *Pseudomonas chlororaphis* PA23 biocontrol revealed through proteomic and phenotypic analysis

**DOI:** 10.1186/1471-2180-14-94

**Published:** 2014-04-17

**Authors:** Natasha Klaponski, Carrie Selin, Kelly Duke, Vic Spicer, Dilantha WG Fernando, Mark F Belmonte, Teresa R de Kievit

**Affiliations:** 1 Department of Microbiology, University of Manitoba, R3T 2N2 Winnipeg, MB, Canada; 2 Department of Physics and Astronomy, University of Manitoba, R3T 2 N2 Winnipeg, MB, Canada; 3 Department of Plant Science, University of Manitoba, R3T 2 N2 Winnipeg, MB, Canada; 4 Department of Biological Science, University of Manitoba, R3T 2 N2 Winnipeg, MB, Canada

**Keywords:** Antifungal, Biocontrol, Chitinase, Motility, Phenazine, *Pseudomonas*, Siderophore, Transcriptional regulator

## Abstract

**Background:**

*Pseudomonas chlororaphis* strain PA23 is a biocontrol agent capable of suppressing the fungal pathogen *Sclerotinia sclerotiorum*. This bacterium produces the antibiotics phenazine and pyrrolnitrin together with other metabolites believed to contribute to biocontrol. A mutant no longer capable of inhibiting fungal growth was identified harboring a transposon insertion in a gene encoding a LysR-type transcriptional regulator (LTTR), designated *ptrA* (*Pseudomonas* transcriptional regulator). Isobaric tag for relative and absolute quantitation (iTRAQ) based protein analysis was used to reveal changes in protein expression patterns in the *ptrA* mutant compared to the PA23 wild type.

**Results:**

Relative abundance profiles showed 59 differentially-expressed proteins in the *ptrA* mutant, which could be classified into 16 clusters of orthologous groups (COGs) based on their predicted functions. The largest COG category was the unknown function group, suggesting that many yet-to-be identified proteins are involved in the loss of fungal activity. In the secondary metabolite biosynthesis, transport and catabolism COG, seven proteins associated with phenazine biosynthesis and chitinase production were downregulated in the mutant. Phenotypic assays confirmed the loss of phenazines and chitinase activity. Upregulated proteins included a lipoprotein involved in iron transport, a flagellin and hook-associated protein and four proteins categorized into the translation, ribosome structure and biogenesis COG. Phenotypic analysis revealed that the mutant exhibited increased siderophore production and flagellar motility and an altered growth profile, supporting the proteomic findings.

**Conclusion:**

PtrA is a novel LTTR that is essential for PA23 fungal antagonism. Differential protein expression was observed across 16 COG categories suggesting PtrA is functioning as a global transcriptional regulator. Changes in protein expression were confirmed by phenotypic assays that showed reduced phenazine and chitinase expression, elevated flagellar motility and siderophore production, as well as early entrance into log phase growth.

## Background

*Pseudomonas chlororaphis* strain PA23 is a biocontrol agent able to protect canola from stem rot disease caused by the fungus *Sclerotinia sclerotiorum* (Lib.) de Bary [1,2]. This bacterium produces a number of compounds including phenazine 1-carboxylic acid (PCA), 2-hydroxyphenazine (2-OH-PHZ), pyrrolnitrin, protease, lipase, chitinase and siderophores, some of which have been shown to contribute to fungal antagonism [3-5]. Public concern over the use of chemical pesticides together with the potential for acquiring resistance to these compounds has led to renewed interest in bacterial antagonists, such as PA23, for biocontrol. Despite demonstrating excellent disease control in the greenhouse, many biocontrol agents suffer from inconsistent performance in the field [6-8]. Poor field performance is likely due, at least in part, to variable expression of genes and gene products required for disease suppression. It is essential, therefore, to elucidate the molecular mechanisms mediating PA23 biocontrol so that production of the pathogen-suppressing factor(s) can be optimized in the environment.

In *Pseudomonas* spp. that act as biocontrol agents, expression of disease-suppressive metabolites is controlled by a multi-tiered network of regulation. One of the key regulatory elements is the GacS/GacA two-component signal transduction system, comprised of the sensor kinase GacS and its cognate response regulator GacA [9]. In many pseudomonads, including PA23, a mutation in *gacS* or *gacA* leads to a loss of fungal antagonism [4,9]. Working in concert with GacS/GacA is the Rsm system which consists of RsmA-like repressor proteins and untranslated regulatory RNAs. The repressor proteins act post-transcriptionally by binding to the ribosome-binding site (RBS) in target mRNA [10]. The regulatory RNAs antagonize repression by titrating out the RsmA-like proteins, rendering the RBS of target genes accessible to the translational machinery [10]. Additional regulatory elements that oversee production of PA23 antifungal metabolites include the PhzR/PhzI quorum-sensing (QS) circuit [11], the stationary phase sigma factor RpoS [12], a regulator of RpoS called PsrA [13], and a global stress response system known as the stringent response [12]. Substantial interaction occurs between the regulators themselves, which adds to the complexity of the regulatory hierarchy [11-13].

Through transposon mutagenesis, a PA23 mutant was identified that exhibited a complete loss of antifungal activity, similar to what is observed for a *gac* mutant [4,13]. Sequence analysis revealed that the interrupted gene, designated *ptrA* (Pseudomonas transcriptional regulator), encodes a protein belonging to the LysR-type transcriptional regulator (LTTR) family. LTTRs can act as either activators or repressors and are known to control a diverse range of metabolic functions including cell invasion and virulence, QS, oxidative stress, and amino acid metabolism [14]. Given the remarkably complex regulatory network that oversees the production of antifungal compounds, the aim of the current study was to understand the global impact of the *ptrA* mutation on PA23 protein expression. Using the isobaric tag for relative and absolute quantitation (iTRAQ) technique, 59 proteins were found to be differentially expressed in the *ptrA* mutant compared to the wild type. Changes in protein expression were confirmed by phenotypic assays that showed reduced phenazine and chitinase expression, elevated flagellar motility and siderophore production, as well as early entrance into the logarithmic growth phase.

## Results and discussion

### Isolation of a *Pseudomonas chlororaphis* PA23 mutant deficient in antifungal activity

**Figure 1:**
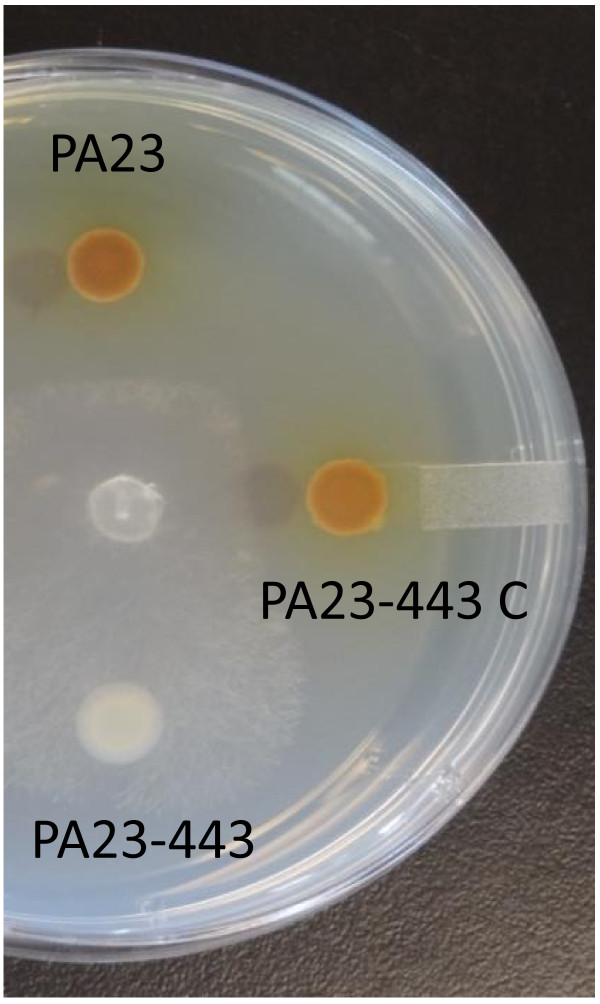
**Antifungal activity of PA23 and derivative strains against *Sclerotinia sclerotiorum*.** Note that the presence of plasmid-borne *ptrA* is able to restore antifungal activity in PA23-443.

**Figure 2:**
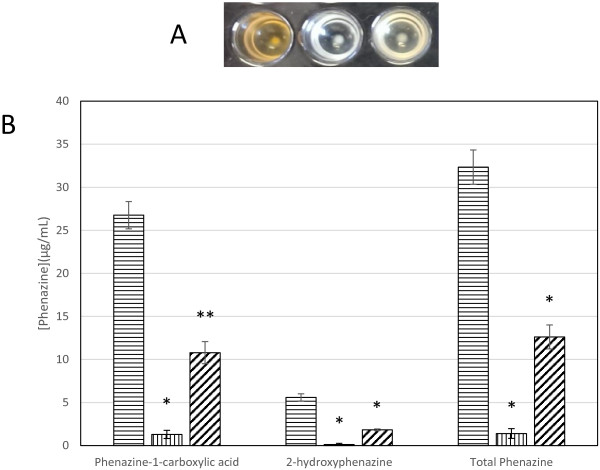
**Phenazine production in PA23, PA23-443, and PA23-443 harboring *ptrA in trans. *Panel A**. Color development of overnight cultures grown in M9 minimal media supplemented with 1 mm MgSO_4_ and 0.2% glucose. Left to right; PA23 (pUCP22), PA23-443 (pUCP22), PA23-443 (*ptrA*-pUCP22). **Panel B**. Quantitative phenazine analysis of cells grown in M9 minimal media supplemented with 1 mm MgSO_4_ and 0.2% glucose. Horizontal lines; PA23 (pUCP22), vertical lines; PA23-443 (pUCP22), diagonal lines; PA23-443 (*ptrA*-pUCP22). Total phenazine: phenazine-1-carboxylic acid + 2-hydroxy-phenazine. *; P < 0.0001, **; p < 0.0002.

Approximately 4000 transconjugants were screened in radial diffusion plate assays to identify mutants displaying increased or decreased antifungal activity compared to the wild type. One mutant was identified, PA23-443, that exhibited no antifungal activity and was white in colour, indicating a loss of phenazine production [5] (Figures 1 and 2B). DNA flanking the Tn exhibited 89% identity at the amino acid level to a *Pseudomonas fluorescens* LTTR [Genbank: AAY90576]. The newly identified gene was designated *ptrA*. To verify that the phenotype of PA23-443 was due to *ptrA* inactivation, the *ptrA* gene was PCR amplified and cloned into pUCP22 for complementation. The presence of pUCP22-*ptrA* restored antifungal activity to that of the wild type (Figure 1).

Sequence analysis revealed that the site of Tn insertion lies 803 bp downstream of the PtrA translational start (data not shown), which is predicted to disrupt the co-inducer recognition/response domain [15]. Previous studies of the LTTRs NodD and NahR revealed that mutations in this region result in a co-inducer-independent phenotype which affects DNA binding and thus the activation/repression properties of the proteins [14,15]. Directly downstream of *ptrA* but in the opposite orientation lies a gene encoding a protein that is 99% identical at the amino acid level to a DoxX-family protein found in *P. chlororaphis* subsp. *aurantiaca* PB-St2 [Genbank accession #WP_023968058]. Based on sequence similarity, DoxX could be involved in pathways related to elemental sulfur oxidation [16]. Immediately upstream of *ptrA,* in the opposite orientation, lies a gene encoding a short-chain dehydrogenase (*scd*). Short-chain dehydrogenases are part of a superfamily of enzymes designated as the NAD(H)- or NADP(H)-dependent short-chain dehydrogenases/reductases (SDRs). The SDRs comprise a very large grouping of biologically important proteins found in virtually all forms of life [17]. At present, it is unclear whether the genes upstream and downstream of *ptrA* play a role in regulation.

Through blastn analysis, *ptrA* homologs were found within the genomes of several *Pseudomonas* species, with the highest degree of nucleotide identity exhibited by *Pseudomonas* sp. UW4 (85%), followed by *Pseudomonas protegens* strains Pf-5 (84.7%) and CHA0 (84.7%), *Pseudomonas fluorescens* strains Pf0-1 (84.5%) and F113 (82.5%), *Pseudomonas brassicacearum* subsp. *brassicacearum* NFM421 (82.4%), *Pseudomonas poae* RE*1-1-14 (79.3%), and *Pseudomonas resinovorans* NBRC 106553 (76.1%) [18]. Collectively, our findings indicate that PtrA is a newly identified regulator of PA23 biocontrol, and homologs of this regulator are present in a number of *Pseudomonas* species.

### Differential protein expression between the PA23 wild type and the *ptrA* mutant

**Figure 3:**
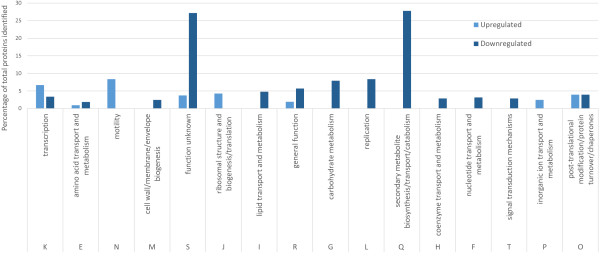
**Differentially expressed proteins in mutant PA23-443 compared to the PA23 wild type.** Fifty-nine proteins were found to be differentially regulated and they were classified into 16 clusters of orthologous groups based on their predicted function.

PtrA belongs to the LTTR family, which is the largest known family of prokaryotic DNA binding proteins [14]. LTTRs can function as either repressors or activators for single or operonic genes. Furthermore, these regulators may be divergently transcribed from their target genes or may control expression of numerous genes scattered about the chromosome [14]. In PA23, expression of antifungal metabolites is governed by a complex network of regulatory elements and substantial interaction occurs between the regulators themselves [4,11-13]. To understand the global impact of the *ptrA* mutation on PA23 physiology, iTRAQ proteomic analysis was carried out to reveal proteins that were differentially expressed between the PA23 wild type and the *ptrA* mutant. A total of 771 proteins were matched to proteins found within the *P. chlororaphis* gp72 reference genome [19]. Fifty nine of these proteins were differentially expressed between the two strains, exhibiting a vector difference (V_diff_) greater than or equal to +1.65 and less than or equal to −1.65, corresponding to proteins in the upper or lower 10% of the population distribution (Table 1). The 59 proteins could be classified into 16 clusters of orthologous groups (COGs) based on their predicted function. Figure 3 summarizes the classification of the identified proteins, indicating significant up- or downregulation of protein expression. The largest COG category was the unknown function group, suggesting that many yet-to-be-identified proteins play a role in the loss of biocontrol exhibited by PA23-443.

**Table 1:** Differentially expressed proteins in mutant PA23-443 compared to the PA23 wild type

**COG Category**	**Locus Tag**	**Predicted Function**	**Fold Change**^**a**^	**V**_**diff **_**Score**
Amino acid transport and metabolism	MOK_00491	4-aminobutyrate aminotransferase and related aminotransferases	1.59	2.24
	MOK_03651	Monoamine oxidase	−2.39	−2.7
	MOK_04019	ornithine carbamoyltransferase	−1.48	−1.67
Nucleotide transport and metabolism	MOK_04929	hypothetical protein	−3.13	−2.54
Carbohydrate transport and metabolism	MOK_03378	Chitinase	−3.30	−3.76
	MOK_05029	Glucose/sorbosone dehydrogenases	−1.68	−2.04
	MOK_05478	Chitinase	−2.61	−1.66
Lipid transport and metabolism	MOK_04573	Acyl dehydratase	−2.16	−2.42
Translation, ribosomal structure and biogenesis	MOK_00565	Translation elongation factor P (EF-P)/translation initiation factor 5A (eIF-5A)	1.61	1.94
	MOK_01324	ribosomal protein L32	2.33	2.77
	MOK_02337	aspartyl/glutamyl-tRNA(Asn/Gln) amidotransferase, C subunit	2.09	1.7
	MOK_04471	ribosomal protein S19, bacterial/organelle	1.49	1.7
Transcription	MOK_02056	cold shock domain protein CspD	−2.31	−1.81
	MOK_02888	Cold shock proteins	2.30	2.44
	MOK_03359	Cold shock proteins	1.26	1.65
Replication, recombination and repair	MOK_00606	competence protein ComEA helix-hairpin-helix repeat region	−2.78	−3.04
Cell wall, membrane and envelope biogenesis	MOK_05137	Outer membrane protein and related peptidoglycan-associated (lipo)proteins	−1.65	−1.79
Cell motility	MOK_01499	Flagellin and related hook-associated proteins	2.71	3.26
Post-translational modification, protein turnover and chaperones	MOK_00750	monothiol glutaredoxin, Grx4 family	1.20	1.81
	MOK_01830	peroxiredoxin, OsmC subfamily	−2.61	−2.69
	MOK_05742	Peroxiredoxin	−1.84	−1.78
	MOK_05953	Peptidyl-prolyl cis-trans isomerase (rotamase) - cyclophilin family	2.00	1.73
Inorganic ion transport and metabolism	MOK_05447	Predicted periplasmic lipoprotein involved in iron transport	1.42	1.73
Secondary metabolites biosynthesis, transport and catabolism	MOK_01048	Phenazine biosynthesis protein A/B.	−4.22	−4.58
	MOK_01049	Phenazine biosynthesis protein A/B.	−3.25	−4.26
	MOK_01053	phenazine biosynthesis protein PhzF family	−1.19	−2.1
	MOK_01054	Pyridoxamine-phosphate oxidase	−1.25	−2.18
	MOK_01055	Aromatic ring hydroxylase	−2.45	−2.43
General function prediction only	MOK_01152	Predicted periplasmic or secreted lipoprotein	−2.29	−2.42
	MOK_02985	intracellular protease, PfpI family	1.67	1.93
	MOK_03813	Predicted O-methyltransferase	−2.12	−1.73
	MOK_05714	Serine protease inhibitor ecotin	−1.33	−1.65
Function unknown	MOK_00258	Protein of unknown function (DUF3313).	−1.81	−2.03
	MOK_00808	hypothetical protein	−8.28	−7.73
	MOK_01097	hypothetical protein	−2.10	−2.22
	MOK_01302	hypothetical protein	−1.32	−2.08
	MOK_01398	hypothetical protein	−2.04	−2.19
	MOK_01832	Protein of unknown function (DUF1161).	−1.14	−1.94
	MOK_02425	Sigma 54 modulation protein/S30EA ribosomal protein.	1.36	2.22
	MOK_02468	poly(hydroxyalkanoate) granule-associated protein	−2.70	−3.66
	MOK_02469	poly(hydroxyalkanoate) granule-associated protein	−1.75	−2.32
	MOK_03057	Uncharacterized protein conserved in bacteria	−1.86	−2.29
	MOK_03064	type VI secretion protein, VC_A0107 family	−2.87	−3.14
	MOK_03065	type VI secretion protein, EvpB/VC_A0108 family	−2.72	−3.02
	MOK_03231	outer membrane porin, OprD family.	1.49	1.8
	MOK_03379	Uncharacterized protein conserved in bacteria	−4.52	−5.06
	MOK_03717	hypothetical protein	−5.36	−6.81
	MOK_03859	hypothetical protein	−2.60	−2.27
	MOK_04005	Protein of unknown function (DUF3613).	−2.39	−2.06
	MOK_04318	Predicted integral membrane protein	−1.80	−2.21
	MOK_04378	Putative phospholipid-binding domain./LysM domain.	−2.22	−3.47
	MOK_04746	hypothetical protein	−2.29	−2.71
	MOK_04755	hypothetical protein	−3.36	−3.84
	MOK_05477	Uncharacterized protein conserved in bacteria	−2.09	−1.41
	MOK_05648	hypothetical protein	−4.51	−4.7
	MOK_05758	hypothetical protein	−4.00	−4.19
	MOK_06084	Iron-sulfur cluster assembly accessory protein	1.72	1.73
	MOK_06136	hypothetical protein	−5.20	−5.37
Signal transduction mechanisms	MOK_04087	Putative Ser protein kinase	−1.38	−2.06

Proteins with V_diff_ ≥ +1.65 or V_diff_ ≤ −1.65, corresponding to proteins expressed in the upper or lower 5% of the population distribution are shown.

^a^log_2_(tag_115_/tag_117_).

### PtrA regulates phenazine production in PA23

The secondary metabolite biosynthesis, transport and catabolism COG category represented the next largest grouping (Table 1). Initially, two of the proteins (MOK_01048, MOK_01053) were classified under the general function category and one protein (MOK_01054) was categorized under the transport and metabolism grouping. Upon further investigation, the locus tags indicated that they are part of the phenazine biosynthetic operon, leading to their reclassification into the secondary metabolite biosynthesis COG.

The phenazine operon has been well characterized in many pseudomonads, with *phzABCDEFG* comprising the core biosynthetic locus [20]. In this study, proteins with locus tags MOK_01048 and MOK_01049, identified as phenazine biosynthesis protein A/B, were significantly downregulated (Table 1). All phenazine-producing pseudomonads have an adjacent and nearly identical copy of the *phzB* gene, termed *phzA*[20]. PhzA catalyzes the condensation reaction of two ketone molecules in the phenazine biosynthesis pathway [20]. PhzF (identified as MOK_01053 in this study) works as an isomerase, converting *trans*-2,3-dihydro-3-hydroxyanthranilic acid (DHHA) into 6-amino-5-oxocyclohex-2-ene-1-carboxylic acid prior to the condensation reaction catalyzed by the PhzA/B proteins [20]. *phzG* encodes an FMN-dependent pyridoxamine oxidase (identified as MOK_01054 in this study), which is hypothesized to catalyze the conversion of DHHA to 5,10-Dihydro-PCA [21]. In some pseudomonads, genes downstream of the core biosynthetic operon are required for generation of phenazine derivatives [22-24]. In *P. chlororaphis* 30–84, for example, *phzO* lies downstream of the core operon; PhzO is an aromatic hydroxylase that catalyzes the conversion of PCA into 2-OH-PHZ [23]. More recently, in *P. chlororaphis* gp72, the *phzO* gene was shown to convert PCA into 2-OH-PHZ through a 2-OH-PCA intermediate [25]. Like other *P. chlororaphis* strains, PA23 produces 2-OH-PHZ and we believe the downregulated aromatic ring hydroxylase (MOK_01055) is PhzO. Therefore, in the absence of a functional *ptrA* gene, four of the core phenazine biosynthetic enzymes (PhzA, PhzB, PhzF, PhzG) and one aromatic ring hydroxylase (PhzO) are significantly downregulated. The fact that PtrA plays a critical role in regulating *phz* expression was not surprising considering the lack of orange pigment produced by the *ptrA* mutant (Figures 1 and 2A). Reduced phenazine expression was further substantiated by quantitative assays. As illustrated in Figure 2B, there is a 15-fold decrease in phenazine production in PA23-443 compared to the PA23 wild type. When *ptrA* was expressed *in trans*, some restoration of phenazine production was achieved.

### Chitinase production is under PtrA control

Our iTRAQ proteomic results showed that two chitinase enzymes (MOK_03378 and MOK_05478) were significantly downregulated in the PA23-443 mutant (Table 1). These results were supported by chitinase assays, which clearly indicated no detectable enzyme activity in the *ptrA* mutant (Table 2). Addition of plasmid-borne *ptrA* elevated chitinase activity close to that of the wild type (Table 2). Collectively our findings indicate that *ptrA* is necessary for chitinase production. The LTTR, ChiR, has been previously shown to indirectly regulate all chitinases produced in *Serratia marcescens* 2170 [26]. Proteomic analysis of a *P. aeruginosa gacA* mutant revealed that chitinase (ChiC) and a chitin-binding protein (CbpD) were decreased 8-fold and 2.2-fold respectively, as compared to the wild type [27].

**Table 2:** **Chitinase activity of *P. chlororaphis *** **strain PA23 and derivative strains**

**Strain**	**Chitinase Activity (A**_550_*min^−1^*mg total protein^**−1**^**)**	
	**Early stationary phase**^**a**^	**Late stationary phase**^**a**^
PA23 (pUCP22)	0.11 (0.03)	0.12 (0.004)
PA23-443 (pUCP22)	0.0 (0.0)^b^	0.0 (0.0)^c^
PA23-443 (*ptrA*-pUCP22)	0.10 (0.03)^d^	0.11 (0.01)^e^

^a^Mean (standard deviation) of enzyme activity of three replicates.

^b^Significantly different from wild type (P < 0.005).

^c^Significantly different from wild type (P < 0.0001).

^d^Not significantly different from wild type.

^e^Significantly different from wild type (P < 0.05).

### Siderophore production is upregulated in PA23-443 compared to the PA23 wild type

In the *ptrA* mutant, a lipoprotein involved in iron transport (MOK_05447) was found to be significantly upregulated (Table 3). This finding prompted us to explore whether the mutant exhibited elevated siderophore expression. Siderophores are thought to contribute to biocontrol by sequestering iron, thereby restricting pathogen growth. Following 24 hours growth on CAS agar plates, mutant PA23-443 showed a 3-fold increase in the size of the orange halo surrounding the colony, indicating increased siderophore production compared to the wild type (Table 3). As expected, overexpression of *ptrA* restored the wild-type phenotype. Since the *ptrA* mutant expresses significantly increased levels of siderophore but exhibits a complete loss of antifungal activity, it is clear that elevated siderophore expression alone is not sufficient for *S. sclerotiorum* control.

**Table 3:** **Siderophore production by *P. chlororaphis *** **PA23, PA23-443 and PA23-443 harboring *ptrA *** **in *trans***

**Strain**	**Zone of orange halo**^**a**^
PA23 (pUCP22)	0.5 (0.0)
PA23-443 (pUCP22)	1.6 (0.2)^b^
PA23-443 (*ptrA*-pUCP22)	0.6 (0.2)^c^

^a^Mean (standard deviation) of orange haloes (mm) surrounding colonies on CAS agar. Five replicates were examined.

^b^Significantly different from the wild type (p < 0.0001).

^c^Not significantly different from the wild type.

### Loss of *ptrA* results in early entry into stationary phase

**Figure 4:**
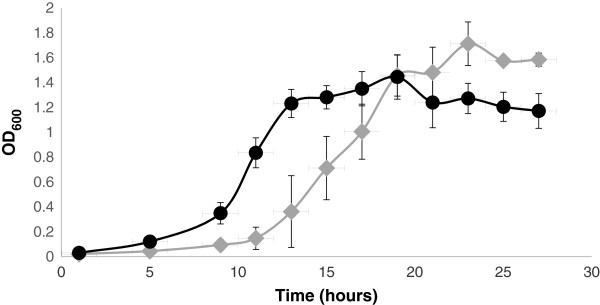
**Growth rate analysis of wild-type PA23 and mutant PA23-443.** Cells were grown in M9 minimal media supplemented with 1 mM MgSO_4_ and 0.2% glucose. Spectrophotometric optical densities were taken at 600 nm. Diamonds; PA23wt, circles; PA23-443.

We observed significant upregulation of proteins involved in translation, ribosomal structure and biogenesis in the *ptrA* mutant (Table 1). These proteins include a translation elongation factor (MOK_00565), a tRNA amidotransferase (MOK_02337) and ribosomal proteins L32 and S19 (MOK_01324 and MOK_04471, respectively) which make up structural components of both the large and small ribosomal subunits of the 70S ribonucleoprotein complex [28] (Table 1). To determine whether PA23-443 exhibited an altered pattern of growth compared to the wild type, growth rate analysis was undertaken. As depicted in Figure 4, the mutant enters the logarithmic (log) growth phase around hour 8, which starts to plateau by hour 13. Conversely, the PA23 wild type does not enter log phase until hour 11, ending with entrance into early stationary phase at 19 hours of growth. Another interesting difference observed was the maximum population density achieved. The PA23 wild type consistently reached a higher OD_600_ in stationary phase compared to PA23-443 (Figure 4). A similar altered pattern of growth has been observed for *gacS* mutants of PA23 and 30–84 which exhibit a shorter lag phase and earlier entry into logarithmic growth phase [4,29]. LTTRs have previously been implicated in the regulation of cellular growth factors. For example, the well-studied LTTR OxyR is involved in regulating the expression of various metabolic genes such as tRNA nucleotidyl transferases and synthetases, ribosomal proteins and QS-regulated targets [30].

### PtrA negatively affects motility

Our iTRAQ proteomic data indicated upregulation of the flagellin and related hook-associated protein (MOK_01499) in PA23-443. Further inspection of the locus tags upstream of MOK_01499 also indicated upregulation of proteins FliG (MOK_01489; V_diff_ = +0.72) and FliS (MOK_01496; V_diff_ = +0.66), although this upregulation was not considered significant. The upregulated flagellin and related hook-associated protein, therefore, is likely part of the Fli operon based on its proximity to upstream genes. To verify the results of the proteomic analysis, motility assays were conducted. As outlined in Table 4, swimming (flagellar) motility was almost 3-fold greater in PA23-443 compared to the wild type, indicating that PtrA is having a repressive effect on this phenotype. In a similar fashion, proteomic analysis of a *P. aeruginosa gacA* mutant revealed a 7.5-fold and 8.8-fold increase in expression of a flagellin (FliC) and flagellar-capping protein (FliD), respectively [27]. Introduction of *ptrA in trans* caused a modest reduction in motility, but did not fully restore the wild-type phenotype. It is important to bear in mind that for our complementation studies, multiple copies of the *ptrA* gene were provided rather than a single chromosomal copy. Because LTTRs bind both activation binding sites and regulatory binding sites upstream of target genes [14], the number of copies of the regulator may be of critical importance for proper binding and subsequent regulation of target genes. This observation was noted with complementation studies involving the LTTR OxyR in restoration of rhamnolipid and pyocyanin production in *P. aeruginosa*[31]. When multiple copies of *oxyR* were present in the cell, the wild-type phenotype was not restored; whereas insertion of single chromosomal copy of the LTTR gene resulted in full complementation [31].

**Table 4:** **Motility analysis of *P. chlororaphis *** **strain PA23, PA23-443 and PA23-443 harboring *ptrA *** **in *trans***

**Strain**	**Motility zone diameter (mm) at 48 h**^**a**^
PA23 (pUCP22)	17.0 (0.0)
PA23-443 (pUCP22)	47.5 (0.6)^b^
PA23-443 (*ptrA*-pUCP22)	43.8 (1.6)^b^

^a^Mean (standard deviation) of swim zones from four replicates.

^b^Significantly different from the wild type (p < 0.0001).

### PtrA regulates pyrrolnitrin production in PA23

Based on iTRAQ analysis, a tryptophan halogenase (MOK_04031) was identified under the amino acid transport and metabolism COG category, but was not significantly differentially expressed in the *ptrA* mutant (V_diff_ = −0.24). At locus tag MOK_04033, another chlorinating halogenase was identified in the *P. chlororaphis* gp72 genome, but was not differentially expressed in the *ptrA* mutant. These enzymes are likely *prnA* and *prnC*, forming part of the *prnABCD* pyrrolnitrin biosynthetic operon [32]. Subsequent pyrrolnitrin quantification via HPLC analysis revealed that wild type PA23 produced an average of 3.48 (±0.45) μg of pyrrolnitrin, whereas in the *ptrA* mutant, no pyrrolnitrin was detected. However, when *ptrA* was expressed *in trans* in PA23-443, pyrrolnitrin production was restored to wild-type levels (3.90 ± 0.20 μg). Significant downregulation of pyrrolnitrin expression may not have been identified through iTRAQ analysis as cell samples were taken at the onset of stationary phase. To obtain enough pyrrolnitrin for quantification, cell culture extracts are routinely performed after five days of growth [5]. Thus, there may have been differences in protein expression in late stationary phase that were not detected in our iTRAQ analysis. As pyrrolnitrin has previously been reported as essential for PA23 biocontrol [5], the lack of pyrrolnitrin production by the *ptrA* mutant is likely a major contributor to the loss of antifungal activity.

## Conclusions

In the present study, we describe the characterization of a PA23 derivative with a mutation in a gene encoding a novel transcriptional regulator, designated PtrA. As the mutant is no longer capable of suppressing the fungal pathogen *S. sclerotiorum*, PtrA is essential for PA23 biocontrol. It is apparent that PtrA affects many facets of PA23 physiology. Differential protein expression was observed across 16 different COG categories, indicating that PtrA is likely acting as a global transcriptional regulator. One of the limitations associated with this study stems from the fact that our proteomic analysis was based on the *P. chlororaphis* gp72 reference genome. In the future, the availability of the PA23 genome sequence may allow us to better understand the function of these differentially expressed proteins. In addition, several aspects of PtrA regulation have yet to be revealed, for example, LTTRs are frequently autoregulated and co-inducer molecules profoundly impact binding specificity [15]. We are currently investigating the DNA targets of PtrA transcriptional regulation, including *ptrA* itself. Furthermore, the nature of the PtrA effector and its role in binding has yet to be discovered. It is hoped that by unraveling the complex regulatory hierarchy overseeing production of antifungal compounds, this bacterium can be used in a consistent and predictable manner for suppression of *S. sclerotiorum* in the field.

## Methods

### Bacterial strains and growth conditions

The bacterial strains and plasmids used in this study are listed in Table 5. *Escherichia coli* strains were cultured at 37°C on Lennox Luria Bertani (LB) agar (Difco Laboratories, Detroit, Michigan). *P. chlororaphis* PA23 and its derivatives were cultured at 28°C on LB agar or M9 minimal media supplemented with 1 mM MgSO_4_ and 0.2% glucose. For antifungal assays, bacteria were grown on potato dextrose agar (PDA; Difco). As required, media were supplemented with the following antibiotics: tetracycline (Tc; 15 μg/mL), gentamicin (Gm; 15 μg/mL), ampicillin (Amp; 100 μg/mL) for *E. coli,* and rifampicin (Rif; 25 μg/mL), Tc (15 or 100 μg/mL), Gm (25 μg/mL), piperacillin (30 or 500 μg/mL) for *P. chlororaphis.* All antibiotics were obtained from Research Products International Corp. (Mt. Prospect, Illinois).

**Table 5:** Bacterial strains, plasmids and primers used in this study

**Strain/plasmid/primer**	**Relevant genotype or phenotype**	**Source or reference**
***P. chlororaphis*** PA23	Phz^+^Rif^R^ wild type (soybean plant isolate)	[1]
PA23-443	Phz^−^ Rif^R^*ptrA*::Tn*5-*OT182 genomic fusion	This study
***E. coli***		
DH5α	*supE44* Δ*lacU169* (φ80 *lacZ*ΔM15) *hsdR17 recA1 endA1 gyrA96 thi-1 relA1*	Gibco
SM10	Mobilizing strain; RP4 *tra* genes integrated in chromosome; Km^R^ Tc^R^	[33]
		
**Plasmids**		
pOTI82	pSUP102(GM)::Tn*5*-OT182 Cm^R^ Gm^R^ Amp^R^ Tc^R^	[34]
pOT182-443 (*Xho*I)	pOT182 containing *ptrA*::Tn*5*-OT182 genomic fusion	This study
pCR2.1TOPO	Cloning vector for PCR products	Invitrogen
pUCP22	Broad-host-range vector; IncP OriT, Amp^R^ Gm^R^	[35]
pUCP22-*ptrA*	pUCP22 containing ptrA from *P. chlororaphis* PA23	This study
**Primers**		
ptrA-F	5′-gggaaccggcttatagcca-3′	This study
ptrA-R	5′-atccagttgctggagcgtatt-3′	This study
TNP5-FORWARD	5′-accatttcaacggggtctcac-3′	[4]
TNP5-REVERSE	5′-tgactccatgtgacctccta-3′	[4]
Tn5-ON82	5′-gatcctggaaaacgggaaagg-3′	[4]
Tn5-OT182 right	5′-atgttaggaggtcacatg-3′	[4]

### PCR

Polymerase Chain Reaction (PCR) was performed under standard conditions as suggested by Invitrogen Life Technologies data sheets supplied with their *Taq* polymerase.

### Nucleic acid manipulation

Cloning, purification, electrophoresis, and other manipulations of nucleic acid fragments and constructs were performed using standard techniques [36]. To clone the PA23 *ptrA* gene, oligonucleotide primers ptrA-F and ptrA-R were used to amplify a 2.2-kb product which was cloned into vector pCR2.1-TOPO following manufacturer’s instructions. The 2.2-kb *ptrA* insert was then excised with *Xba*I and *Bam*HI and cloned into the same sites of pUCP22, generating pUCP22-*ptrA*.

### Tn*5*-OT182 transposon mutagenesis

Bacterial conjugations were performed to introduce Tn*5*-OT182 into *P. chlororaphis* PA23 by biparental mating following the method of Lewenza et al., [37]. For each mating, 5–10 Tc^R^ colonies were screened by PCR to ensure that transconjugants contained a Tn*5* insertion using TNP5-FORWARD and TNP5-REVERSE primers. To determine the site of Tn*5*-OT182 insertion, rescue cloning was performed following previously described methods [37].

### Sequence analysis and nucleotide accession number

Plasmids isolated from Tc^R^*Xho*I clones were sent for sequencing using oligonucleotide primer Tn*5*-ON82, which anneals to the 5′ end of Tn*5*-OT182. *BamH*I or *Cla*I rescue plasmids were sequenced using primer Tn*5*-OT182 right, which anneals to the 3′ end of the transposon. All sequencing was performed at the University of Calgary Core DNA Services facility. Sequences were analyzed using BLASTn and BLASTx databases (http://blast.ncbi.nlm.nih.gov/Blast.cgi?CMD=Web&PAGE_TYPE=BlastHome). The GenBank accession number for the *P. chlororaphis* PA23 *ptrA* gene sequence is EF054873.

### Antifungal assays

Radial diffusion assays to assess fungal inhibition against *S. sclerotiorum* in vitro were performed with wild-type PA23, mutant PA23-443 and PA23-443 harboring the *ptrA* gene *in trans* according to previously described methods [4]. Five replicates were analyzed for each strain and assays were repeated three times.

### Proteomic analysis

Wild-type PA23 and mutant PA23-443 cells were grown as duplicate samples. At the point when cultures were just entering stationary phase (OD_600_ = 1.2), they were centrifuged at 10,000 × g for 10 minutes at 4°C, and pellets were washed three times in PBS buffer and frozen at −80°C. Further sample preparation and iTRAQ labelling was carried out at the Manitoba Centre for Proteomics and Systems Biology. Briefly, 100 μg protein samples were mixed with 100 mM ammonium bicarbonate, reduced with 10 mM dithiothreitol (DTT) and incubated at 56°C for 40 min. Samples were then alkylated with 50 mM iodoacetamide (IAA) for 30 min at room temperature in the dark. Addition of 17 mM DTT was used to quench excess IAA, and proteins were digested with sequencing-grade trypsin (Promega, Madison, WI, USA) overnight. Dried samples were then desalted with 0.1% trifluoroacetic acid and subjected to two-dimensional high-performance liquid chromatography (2D-HPLC)-mass spectrometry (MS) according to previously described methods [38].

### Database search and protein identification

2D-HPLC-MS/MS spectra data from three independent runs were analyzed using ProteinPilot (v2.0.1, Applied Biosystems/MDS Sciex, Concord, ON, Canada) which employs the Paragon™ algorithm. Searches were performed against the *P. chlororaphis* strain gp72 reference genome. Reporter ion iTRAQ tags were labelled as follows: tags 114 and 115 to replicates of wild-type PA23 grown to early stationary phase, and tags 116 and 117 to replicates of mutant PA23-443 grown to early stationary phase. Results were reported as Z-scores, the log2 of the ratio among replicates (Z0 = tag_116_/tag_114_; Z1 = tag_117_/tag_115_; Z2 = tag_115_/tag_114_; Z3 = tag_117_/tag_116_). Peptide Z-scores values were histogrammed (Z0, Z1) to determine the overall population distribution. Further statistical analysis was performed according to the methods outlined in Rydzak et al., [38]. Briefly, V_diff_ scores were assigned to allow the determination of statistical significance of protein expression ratios between both the wild-type and mutant samples while also taking into account the variation between biological replicates. Plotted Z-scores were transformed into vector values, allowing comparison between points (Z0,Z1) and (Z2,Z3). Differences between magnitudes of the vector values from the origin to points (Z0,Z1) and (Z2,Z3) were adjusted to the widths of the peptide population distributions. Direction of the vector values (+or -) were assigned based on the angle subtended by the vector value from the origin to point (Z0,Z1). A V_diff_ value greater than or equal to +1.65 and less than or equal to −1.65 corresponds to proteins expressed in the upper or lower 10% of the population distribution [38]. Functional classification of proteins was carried out using the Integrated Microbial Genomes (IMG) database (http://img.jgi.doe.gov/cgi-bin/w/main.cgi) against the *P. chlororaphis* strain gp72 genome.

### Growth curve analysis

Cultures of wild-type PA23 and mutant PA23-443 were inoculated at a starting optical density (OD) _600_ of 0.01 and grown in M9 minimal media (1 mM MgSO_4_; 0.2% glucose). OD_600_ readings were taken at 1 hour, 5 hours and 9 hours, followed by readings every 2 hours until 27 hours of growth. Triplicate samples were analyzed.

### Chitinase assay

PA23 and derivative strains were assayed for chitinase production during early stationary and late stationary phases following the methods outlined by Wirth and Wolf [39]. Briefly, cultures were grown to the desired growth phase in M9 minimal media (1 mM MgSO_4_; 0.2% glucose) and 250 μL aliquots of each of cell-free supernatant, 0.1 M NaOAc, pH 5.2 and carboxymethyl-chitin-Remazol brilliant violet aqueous solution (Loewe Biochemica, Germany) were incubated for 1 hour at 37°C. The reaction was stopped by the addition of 250 μL 1 M HCL. Reaction mixtures were cooled on ice for 10 min and spun at 20,000 × g for 10 min, and the absorbances at 550 nm were recorded. Each experiment was performed in triplicate.

### Flagellar motility analysis

Flagellar (swimming) was monitored according to Poritsanos et al.*,*[4]. Strains were grown overnight in M9 minimal media (1 mM MgSO_4_; 0.2% glucose) and 5 μL was inoculated into the center of 0.3% M9 agar plates. Four replicates were analyzed and the experiment repeated three times.

### Phenazine analysis

Overnight cultures in M9 minimal media (1 mM MgSO_4_; 0.2% glucose) were subjected to phenazine extraction and quantification by UV-visible light spectroscopy at 367 nm and 490 nm for PCA and 2-OH-PHZ, respectively [5]. Phenazine analysis was performed in triplicate.

### Siderophore analysis

Overnight cultures grown in M9 minimal media (1 mM MgSO_4_; 0.2% glucose) were spotted onto CAS media according to the methods outlined in Schwyn and Neilands [40] to analyze siderophore production.

### Pyrrolnitrin analysis

Production of the antibiotic PRN was quanitified according to the methods outlined in [5]. Briefly, 20 mL cultures of PA23 and its derivatives were grown for 5 days in M9 minimal media and PRN was extracted with an equal volume of ethyl acetate. Before extraction, toluene (5 mL) was added to each sample as an internal control. Toluene and PRN UV absorption maxima were recorded at 225 nm with a Varian 335 diode array detector. PRN peaks were detected at 4.7 mins. Samples were analyzed in duplicate.

### Statistical analysis

All statistical analysis was performed using unpaired Students’s *t* test.

### Availability of supporting data

The data sets supporting the results of this article are included within the article.

## Abbreviations

LTTR: LysR-type transcriptional regulator; PtrA: Pseudomonas transcriptional regulator A; iTRAQ: Isobaric tag for relative and absolute quantitation; PCA: Phenazine 1-carboxylic acid; 2-OH-PHZ: 2-hydroxyphenazine; RBS: Ribosome-binding site; QS: Quorum sensing; COG: Clusters of orthologous groups; CAS: Chrome Azurol S; DTT: Dithriothreitol; IAA: iodoacetamide; 2D-HPLC: Two-dimensional high-performance liquid chromatography; MS: Mass spectrometry; Vdiff: Vector difference; OD: Optical density.

## Competing interests

The following patent has been filed:

*ptrA* gene and uses therefore. Inventors: de Kievit, T., Selin, C., and Fernando, D. US patent application # US 12/446,745, filed Feb. 1, 2010 (status: patent pending).

## Authors’ contributions

NK, WGDF, MB and TdK conceived and designed the study. NK drafted the manuscript with input from TdK. NK prepared samples for proteomic analysis; NK, CS and KD performed the phenotypic characterization of the *ptrA* mutant. VS assisted with the proteomic analysis. All authors read and approved the final manuscript.
